# Ophthalmic findings in sheep treated with closantel in Curitiba, Brazil

**DOI:** 10.14202/vetworld.2020.860-864

**Published:** 2020-05-08

**Authors:** Marianna Bacellar-Galdino, Fabiano Montiani-Ferreira, Andre Tavares Somma, Ricardo Guilherme D'Otaviano de Castro Vilani, Ivan Roque de Barros Filho

**Affiliations:** Department of Veterinary Sciences, Federal University of Parana, Curitiba, Brazil

**Keywords:** closantel electroretinography, histopathology, sheep

## Abstract

**Background and Aim:**

Closantel is a widely used anti-parasitic drug that is known to cause ophthalmic problems that lead to blindness. The aim of this study was to investigate the possible electroretinographic changes in sheep that received closantel.

**Materials and Methods:**

Twenty-four 30-day-old Suffolk sheep were split into control group (12 animals) and closantel group (12 animals). The latter group received 15 mg/kg of closantel subcutaneously immediately after the first electroretinography (ERG). The ISCEV protocol was used to perform the ERGs pre-dose (0), 7, and 30 days after treatment. Statistical analyses to compare ERG responses using t-test and analysis of variance were performed (p<0.05). Three months later, the animals were euthanized and the eyes and a part of optic nerve were collected for histopathology. Photography of the retina and optic nerve was taken, and measures of the retinal layers were made and analyzed by paired t-test.

**Results:**

Closantel group showed a significant increase of the mean scotopic a-wave amplitude from 0 to 7 days after closantel administration, using a stimulus of 10,000 mcd.s/m^2^ and a decrease of the mean scotopic and photopic a-wave amplitude (from 7 to 30 days) using the same flash intensity, as well as a decrease in mean photopic b-wave amplitude (from 7 to 30 days) within the group. Control group showed a significant increase of the mean scotopic b-wave implicit time from pre to 30 days after treatment and an increase of the mean scotopic a-wave implicit time from pre to 7 days after treatment, with the stimulus of 10,000 mcd.s/m^2^. This group also showed a decrease in mean photopic b-wave implicit time (from pre to 30 days after treatment), using a stimulus of 10,000 mcd.s/m^2^ and a decrease in mean photopic a-wave implicit time from pre to 30 days after treatment, using a stimulus of 3000 mcd.s/m^2^. The no difference was found in images neither in the measurements of the retina layers.

**Conclusion:**

As observed by ERG responses and the histopathology, a dose of 15 mg/kg of closantel does not significantly affect retinal and optic nerve structures in sheep but the electroretinographic results, however, showed alterations on the phototransduction.

## Introduction

Closantel [N-{5-chlorine-4-[(4-chlorophenol) cyanomethyl]-2-methylphenol}-2-hydroxide 3.5-di iodobenzamide) is an anti-parasite agent that belongs to the group of salicylanilides, commonly used in *Ruminantia* of small sizes, and efficient in treating *Haemonchus contortus, Fasciola hepatica*, and *Oestrus ovis* [1]. This drug is widely connected (>99%) to plasmatic proteins, especially albumin, in such a way that it extends the level of closantel in the plasma, thus protecting sheep from re-infection for up to 28 days [2-4]. There are several reports of intoxication by this drug in bovines [5], caprines [6-9], ovines [10-15], and canines [16], in addition to human beings [17-21]. Among the clinical symptoms reported, the most frequent are ophthalmic problems that lead to blindness.

According to Tsang and Sharma [22], electroretinography (ERG) is an exam that captures the mass electrical response that originates in the retina after a stimulus of light. It is an excellent tool for studying the function of the retina because it is an easy, non-invasive exam widely used in the clinical routine of small animals to help diagnose some illnesses such as progressive retinal atrophy [23] and sudden acquired retinal degeneration [24], and to evaluate the retinal function before a cataract-removal surgery. This exam has been used in several researches, especially those involving drugs that may affect retinal response, causing blindness [25-27].

The aim of this study was to verify if there are electroretinographic and histopathological changes of the retina and optic nerve in sheep treated with closantel.

## Materials and Methods

### Ethical approval

The study was conducted in accordance with the Federal University of Parana (UFPR) Animal Use Ethics committee guidelines (Approval no.030/2008), and all the procedures were carried out pursuant to the Brazilian legislation on animal care and welfare.

### Study period and study location

This study was performed from October 2008 to October 2009 at UFPR Experimental Farm.

### Animals

This study used 24 healthy Suffolk sheep from UFPR Experimental Farm. Animals were approximately 30 days old in the beginning of the experiment. After first assessment (fundoscopy and ERG), they were randomly divided in two groups of 12 animals: (a) Control group and (b) Closanted group that received a dosage of 15 mg/kg of closantel (Zantec®, Biofarm, Jaboticabal, SP) subcutaneously [12].

### ERG

The animals were submitted to indirect fundoscopic exams with the use of a 20-diopter magnifier and ERG, using a portable HMsERG (Ocusciences., Henderson, NV) and ISCEV protocol.

This protocol consists of seven steps. The first three tests (Rods – 10 mcd.s/m^2^, Cones and Rods Pattern – 3000 mcd.s/m^2^, and High intensity Rods and Cones ‒10,000 mcd.s/m^2^) are done after adaptation to the dark (20 min) and the other four (Cones – 3000 mcd.s/m^2^, High intensity Cones – 10,000 mcd.s/m^2^, Pattern Flicker – 3000 mcd.s/m^2^, and High intensity Flicker – 10,000 mcd.s/m^2^) after adaptation to the light (10 min) – such light is beamed by the equipment itself at 30,000 mcd.

The animals were pre-medicated with 0.5 mg/kg of diazepam (Compaz® 5 mg/ml, Cristália, São Paulo, SP) and 0.5 mg/kg of morphine (Dimorf® 10 mg/ml, Cristália, São Paulo, SP), and placed in a dark room for 20 min, with an application of 1 drop of 1% tropicamide ophthalmic preparation (Mydriacyl®, Alcon, São Paulo, SP) in the left eye. After adaptation to the dark, the sheep were anesthetized with 3 mg/kg of propofol (Propovan® 10 mg/ml, Cristália, São Paulo, SP) combined with 1 mg/kg of S(+) ketamine (Ketamine S(+)® 50 mg/ml, Cristália, São Paulo, SP). To maintain the level of anesthesia, two applications of a quarter of the induction dosage of these drugs were done: The first at 8 min and the second at 14 min after induction.

The animals were placed on a rubberized table, laying on their right side. Electrodes were placed as soon as the palpebral movements ceased and there was tolerance to the blepharostat. Next, subdermal electrodes were positioned (FD-E2-24, Grass Technologies, Astro-Medical, Inc., West Warwick, RI). The first electrode is called “ground” and was inserted in the animal’s left axillary area, followed by the “reference,” which was inserted 2.5 cm from the temporal corner of the palpebral rima of the eye to be examined. With the help of a blepharostat, the contact electrode (ERG-*jet*, Nicolet Instruments, Madison, WI) was positioned on the animal’s cornea.

New ERG was performed 1 week (37 days old) after application and 1 month (60 days old) after application, using the same protocol mentioned above. This data were observed by the ERGVIEWER 2.1 software (RetVetCorp, Inc., Columbia, Missouri) and analyzed by the Statview software (SAS Institute Inc. Copyright^©^ 1992-1998) using the paired t-test and ANOVA, using p<0.05.

### Histopathology

All animals were slaughtered after 3 months for the histopathological analysis of the eyes and optic nerve. Eyes and optic nerves were collected without the periorbital, conjunctive, and palpebral excess fat. A dosage of 0.25 mL of 10% solution of formaldehyde in PBS was injected inside the posterior chamber of the eyes, with a 13×4.5 mm needle and a 1 mL syringe, and they were placed in individual recipients with 10% solution of formaldehyde in PBS (1:20) for histopathological examination.

**Figure-1:**
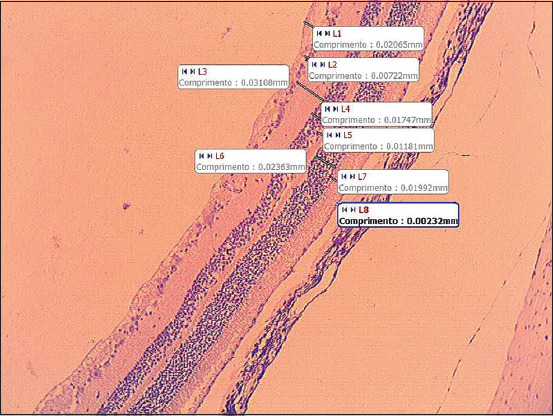
Representative example of the interface of the software Motic Images plus 2.0 (Motic China Group Co. LH 1999-2004). Retina of an ovine control using a 10× objective, showing the measurement of the retina (layers) in millimeters, where L1 – nervous fibers layer; L2 – ganglion cells layer; L3 – inner plexiform layer; L4 – inner nuclear layer; L5 – outer plexiform layer; L6 – outer nuclear layer; L7 – photoreceptor layer; and L8 – retinal pigment epithelium; Comprimento = length.

The specimen was cut lengthwise into four parts and prepared for histologic examination, and then stained using the hematoxylin and eosin technique. Pictures (40× objective) for microscopical analysis and measurements of the layers of the retina (Figure-1) were taken, always 2 mm from the entrance to the optical nerve, as well as pictures of the optical nerve (4×, 10× and 20× objectives), using the Motic Images plus 2.0 software (Motic China Group Co. LH 1999-2004). The data obtained were analyzed statistically by paired t-test.

## Results

**Figure-2:**
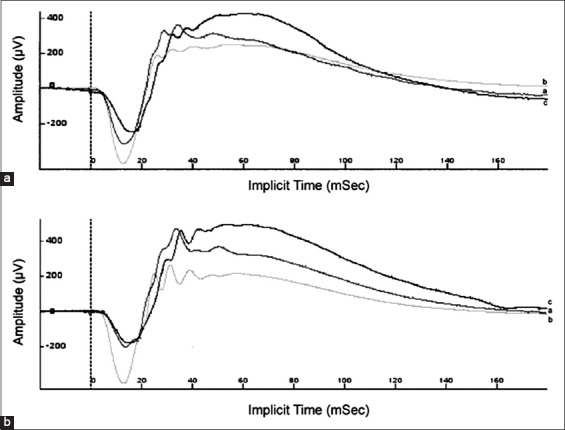
Electroretinographic lines obtained in scotopic test of maximum intensity (10,000 mcd.s/m^2^), (a) 30 days of age – 1^st^ electroretinography (ERG), (b) 37 days of age – 2^nd^ ERG, (c) 60 days of age – 3^rd^ ERG; (a) control group and (b) group treated with closantel.

The group that received closantel had an increase of the implicit time of b-wave in the Flicker tests (30 days old – 22.9±0.66 ms, 37 days old– 23.39±0.39 ms, and 60 days old– 53.6±35.82 ms). In regard to the scotopic test of 10,000 mcd.s/m^2^, there was an increase of amplitude of a-wave comparing 37 and 30 days old (382±150.71 µV; 495.54±120.48 µV, respectively) and a decrease of the amplitude of a-wave when comparing 60 days old (366.41±114.93 µV) to 37 days old (495.54±120.48 µV). Another important change was found in the photopic test with the intensity of 10,000 mcd.s/m^2^: The decrease of amplitudes of a- and b-waves when comparing 37 days old (110.72±14.45 µV; 427.72±55.02 µV) to 60 days old (91.45±25.34 µV; 341±121.95µV) (Figure-2b).

The control group, differently from the group treated with closantel, had an increase of the implicit time of b-wave in the scotopic test with the intensity of 10,000 mcd.s/m^2^ and a decrease of the implicit time of b-wave in the photopic test with the intensity of 10,000 mcd.s/m^2^, all of which variations when comparing the last ERG (60 days) to the first (30 days). During the photopic test with intensity of light of 3000 mcd.s/m^2^, the implicit time of a-wave had a decrease, when comparing 60 days old (11.12±0.6 ms) with 30 days old (11.76±0.72 ms) (Figure-2a). Even though there was statistically significant, these changes are within the normal range for ERG implicit times in sheep [28].

There was no significant difference in the electroretinographic response when comparing the measurements of the two groups in any of the tests.

**Figure-3:**
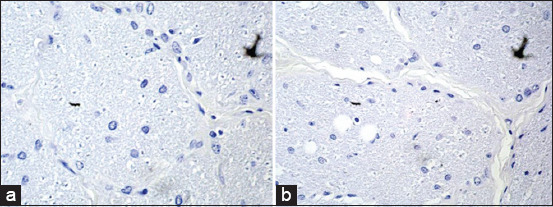
Photomicrography of the optical nerve using a 40× objective, optical nerved stained with hematoxylin and eosin. (a) group treated with closantel and (b) control group.

The dosage used was the highest therapeutic dose recommended. Using this dosage, no microscopic lesions were observed in retinal and optic nerve sections, such as vacuolization of the axons of the inner nuclear layer or other retinal lesions previously described. Regarding the measurement of the retinal layers in the histopathological exam, there was no statistically significant difference (Figure-3).

## Discussion

In vertebrate organisms, the retina is the neural tissue at the back of the eye responsible for converting photons into a signal recognizable by the brain. The three main neurons in this process are photoreceptors, bipolar cells, and ganglion cells [29]. A photon of light is absorbed by the photoreceptor photopigment, and then a series of conformational changes occur. This causes a hyperpolarization of the cell and lead to a decrease in glutamate release at the synaptic terminal. The glutamate reduction or release will stimulate the hyperpolarization or depolarization of the bipolar cells that will send the stimulus to the ganglion cells and then brain [30]. Clinically, the dosage of 15 mg/kg of closantel did not cause blindness in any of the animals involved in this experiment. Likewise, it did not compromise the general function of the retina observed by the ERG exam. However, it caused slight changes in the maturation of speed and the amplitude of responses of some phototransduction mechanisms [15-28,31]. When comparing the amplitude and average implicit time in both groups, there was no significant variation, but if comparing responses within the groups, we can observe that the retina of the group treated with closantel matured differently from the control group.

The group that received closantel had some changes. The main change was the significant increase of amplitude of a-wave in the scotopic test, 7 days after the application of closantel. Even though there was no apparent morphological change in the retina, the slight decrease of the a- and b-wave amplitudes found in the 30-day-old animals’ photopic test suggests that closantel at this dose might interfere somehow with the function of photoreceptors, possibly at the biochemical level of the phototransduction cascade. Therefore, these results also suggest that with higher doses of closantel a more significant interference in photoreceptor function and even retinal lesions would be possible [14].

It is well known that drugs can alter and interfere with retinal function [25-28,31]. The decrease of the a- and b-wave amplitudes found in the 30-day-old animals’ photopic test, compared to the 60-day ERG, might be an effect of closantel in the normal development of cones that were not observed in the evolution of the control group’s electroretinographic response.

Differently, from the histopathologic findings of Cadioli *et al*. [12], Furlan *et al*. [13], and Rivero *et al*. [14] neither central vacuolization of the optic nerve, nor areas of hemorrhage or edema in the retina were observed (Figure-2a). Nevertheless, Furlan *et al*. [13] and Rivero *et al*. [14] used a higher dosage of closantel, which explains the appearance of such wounds. Cadioli *et al*. [12] reported the lesions when a dosage of 15 mg/kg was accidentally used – dosage identical to the one used in the experiment reported herein. Since such lesions were not observed here, we believe that the accidental dosage was understated.

## Conclusion

The use of a dosage of 15 mg/kg of closantel caused neither a permanent change in retinal or optical nerve structure nor a change in vision of the treated animals that would be clinically detectable, which makes its use in sheep relatively safe. The changes found in electroretinographic exams, however, suggest that this drug, in the dosage used, transitorily changes (amplitudes and implicit times) some mechanisms within the phototransduction process that happens in the retina, which seems to cause blindness in toxic dosages.

## Authors’ Contributions

MB designed, conducted the experiment, analyzed data, and prepared the manuscript, FM and IRBF designed, conducted the experiment, and prepared the manuscript, ATS and RDCV conducted *in vivo* experiment and revised manuscript. All authors read and approved the final manuscript.
